# Obstetricians’ perspectives on trial of labor after cesarean (TOLAC) under the two-child policy in China: a cross-sectional study

**DOI:** 10.1186/s12884-021-03559-1

**Published:** 2021-01-28

**Authors:** Zhong-chen Luo, Xu Liu, Anni Wang, Jian-qiong Li, Ze-hong Zheng, Sun Guiyu, Ting Lou, Jin Pang, Xiao-ling Bai

**Affiliations:** 1 School of Nursing, Guizhou Medical University, Guiyang, China; 2 The Nethersole School of Nursing, Faculty of Medicine, The Chinese University of Hong Kong, Hong Kong, China; 3 School of Nursing, Fudan University, Shanghai, China; 4 School of Nursing, Chongqing Three Gorges Medical College, Chongqing, China; 5 Engineering Training Center, Guizhou Minzu University, Guiyang, China; 6 Nursing Department, Guizhou Provincial Peoples Hospital, Guiyang, China; 7 Guizhou Nursing Vocational College, Dazhi Road, Guiyang, 550025 China

**Keywords:** Obstetrician, Trial of labor after cesarean section (TOLAC), Perspective

## Abstract

**Background:**

As the birth policy has been adjusted from one-child-one-couple to universal two-child-one-couple in China, there is an increasing number of women undergoing a second pregnancy after a previous cesarean section (CS). Undertaking an elective repeat CS (ERCS) has been taken for granted and has thus become a major contributor to the increasing CS rate in China. Promoting trial of labor after CS (TOLAC) can reduce the CS rate without compromising delivery outcomes. This study aimed to investigate Chinese obstetricians’ perspectives regarding TOLAC, and the factors associated with their decision-making regarding recommending TOLAC to pregnant women with a history of CS under the two-child policy.

**Methods:**

A cross-sectional survey was carried out between May and July 2018. Binary logistic regression was used to determine the factors associated with the obstetricians’ intention to recommend TOLAC to pregnant women with a history of CS. The independent variables included sociodemographic factors and perceptions regarding TOLAC (selection criteria for TOLAC, basis underlying the selection criteria for TOLAC, and perceived challenges regarding promoting TOLAC).

**Results:**

A total of 426 obstetricians were surveyed, with a response rate of ≥83%. The results showed that 31.0% of the obstetricians had no intention to recommend TOLAC to pregnant women with a history of CS. Their decisions were associated with the perceived lack of confidence regarding undergoing TOLAC among pregnant women with a history of CS and their families (odds ratio [OR] = 2.31; 95% CI: 1.38–1.38); obstetricians’ uncertainty about the safety of TOLAC for pregnant women with a history of CS (OR = 0.49; 95% CI: 0.27–0.96), and worries about medical lawsuits due to adverse delivery outcomes (OR = 0.14; 95% CI: 0.07–0.31). The main reported challenges regarding performing TOLAC were lack of clear guidelines for predicting or avoiding the risks associated with TOLAC (83.4%), obstetricians’ uncertainty about the safety of TOLAC for women with a history of CS (81.2%), pregnant women’s unwillingness to accept the risks associated with TOLAC (81.0%) or demand for ERCS (80.7%), and the perceived lack of confidence (77.5%) or understanding (69.7%) regarding undergoing TOLAC among pregnant women and their families.

**Conclusion:**

A proportion of Chinese obstetricians did not intend to recommend TOLAC to pregnant women with a history of CS. This phenomenon was closely associated with obstetricians’ concerns about TOLAC safety and perceived attitudes of the pregnant women and their families regarding TOLAC. Effective measures are needed to help obstetricians predict and reduce the risks associated with TOLAC, clearly specify the indications for TOLAC, improve labor management, and popularize TOLAC in China. Additionally, public health education on TOLAC is necessary to improve the understanding of TOLAC among pregnant women with a history of CS and their families, and to improve their interactions with their obstetricians regarding shared decision making.

**Supplementary Information:**

The online version contains supplementary material available at 10.1186/s12884-021-03559-1.

## Background

China’s National Maternal and Child Health Statistics showed that the rate of cesarean section (CS) increased from 34.9% in 2014 to 36.7% in 2018 in China [1], which is much higher than the rate recommended by the World Health Organization (i.e., 10–15%) [2]. In 2016, China ended its longstanding one-child per couple policy, and adopted a two-child per couple policy to address the challenge of the ageing population. The number of pregnant women increased sharply, including those with a history of CS. Since the introduction of the two-child policy, women who have become pregnant after a history of CS have been blindly encouraged to undergo an elective repeat CS (ERCS). This approach has been taken for granted and thus has become a major contributor to the increasing CS rate in China [3, 4].

However, ERCS is not the only delivery mode available for pregnant women with a history of CS. Vaginal birth after CS (VBAC) is another option that can reduce the risk of maternal complications, shorten maternal recovery time, improve maternal satisfaction, and be more cost-effective than ERCS [5, 6]. VBAC refers to a vaginal delivery following a successful trial of labor after CS (TOLAC), which is a trial of labor in women with a history of CS regardless of the outcome [7]. Studies have shown that accurate assessments of the risks and benefits of TOLAC and the recommendation of TOLAC to pregnant women with a history of CS have been of vital importance for reducing the CS rate [8, 9]. According to current guidelines, TOLAC is a reasonable choice (taking into account both maternal and infant health) for pregnant women with a history of CS [10, 11]. However, only 13.0–29.3% of pregnant women with a history of CS opt for TOLAC in China [12, 13], while the rate is higher in other countries (ranging from 28 to 82% between 1966 and 2009) [14]. The success rate of TOLAC ranges from 60 to 84% [15–17]. However, TOLAC can fail and can lead to repeated CS, instrumental delivery, and even worse outcomes such as complications (< 1%) including, though very rarely (< 1‰), uterine rupture [10, 18–21].

Various factors should be considered when making a decision about TOLAC, especially in pregnant women with a history of CS. The key factors that influence the adoption of TOLAC are obstetricians’ perceptions about TOLAC/VBAC and the way in which they introduce this delivery mode to pregnant women with a history of CS and their families [22, 23]. However, little is known about obstetricians’ perceptions regarding TOLAC/VBAC for pregnant women with a history of CS and the decision-making process regarding recommending TOLAC to pregnant women with a history of CS in China. Therefore, this study aimed to investigate Chinese obstetricians’ perspectives on TOLAC and to identify the factors associated with recommending TOLAC to pregnant women with a history of CS.

## Methods

### Study design, setting, and eligibility criteria

This was a cross-sectional questionnaire survey conducted between May and July 2018 in China to collect data from a large number of obstetricians. Obstetricians were invited to take part in this study if they: (1) were qualified as physician, with a qualification granted by the National Health Commission (NHC); (2) had helped pregnant women with a history of CS to make antenatal decisions; and (3) consented to take part in this study. Obstetricians working in hospitals that did not offer TOLAC to women with a history of CS were excluded.

### Ethical approval

Ethical approval was obtained from the Medical Ethics Committee of Guizhou Provincial People’s Hospital (no. 2018086) in Guiyang, China. The research conforms to the provisions of the Declaration of Helsinki (1995; as revised in Edinburgh in 2000).

### Data collection

Participants were recruited using a combination of convenience sampling and snowball sampling. To distribute the questionnaire, we used the professional online survey platform Questionnaire Star (Changsha Ranxing Information Technology Co., Changsha, China), which has been used by 33.75 million questionnaire designers to collect a total of 2.334 billion responses. The questionnaire link generated by Questionnaire Star was first sent via WeChat (the social media application with the most users in China) to all obstetricians in Guizhou Provincial People’s Hospital Health Alliance, which consists of 33 hospitals in Guizhou province. We then asked the obstetricians to send the questionnaire link to obstetrician WeChat groups or peers who met the inclusion criteria in their interpersonal circles. To show our appreciation, all participants who finished the survey were offered a “red packet” (a small amount of money) on WeChat as a reward; the distributors also received an additional bonus.

The questionnaire included details on the purposes of our study and the potential risks and benefits, an informed consent form, and the questions. Participants could quit the survey at any time without any consequences by leaving the webpage. To help to identify eligible participants, two questions (i.e., *Are you a clinical obstetrician with a qualification granted by the National Health Commission (NHC)?* and *Are you working in a gynecology and obstetrics department that allows a trial of labor after cesarean to be offered to women with a history of cesarean section?*) were listed at the beginning of the questionnaire. Only participants who answered “Yes” to both questions were allowed to access the rest of the questionnaire.

**Fig. 1:**
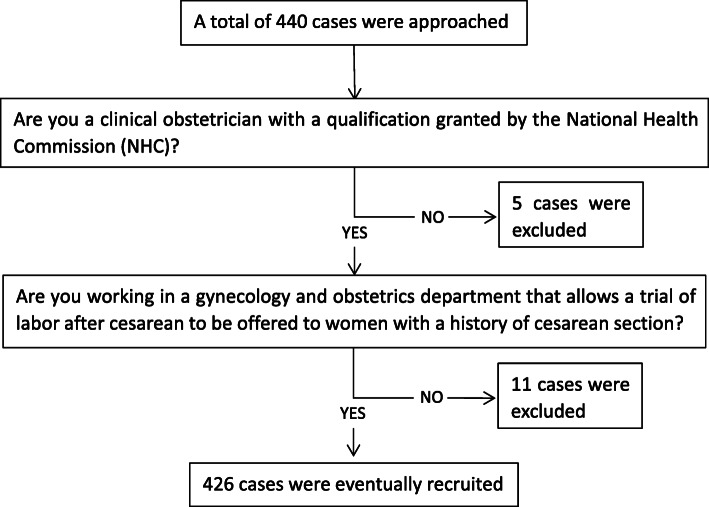
Data collection process diagram

The sample size was determined using a power analysis in the G*Power program [24, 25]. We estimated that at least 337 participants were required based on the following factors: effect size (odds ratio [OR] = 1.5), number of related factors (22), significance level (*p* = 0.05), and power (95%) [8, 10, 11, 26–30]. A total of 440 participants submitted the questionnaire, 14 of who were ineligible and so were excluded from analysis. Missing data is not allowed by the software and a reminder will emerge if any questions are missed by the responders (Fig. 1). The responses of 426 obstetricians, from 62 cities in 23 provinces in China, were regarded as valid. The Questionnaire Star background data showed that the questionnaire had been visited 527 times (including visits when the questionnaire was not ultimately submitted). The actual number of people who visited the questionnaire could be less, as some respondents may have clicked the link more than once (the platform only records the actual number of visits). Therefore, the response rate was ≥83% (Response rate ≥ 426/(527–[440–426]).

### Questionnaire

The questionnaire (***Attachment 1***) was designed according to the existing literature [8, 10, 11, 26–30]. It was in Chinese, had 16 items, and comprised the following sections: i) sociodemographic factors; ii) intention to recommend TOLAC to pregnant women with a history of CS; iii) selection criteria (indications) for TOLAC in clinical practice; iv) basis underlying the selection criteria for TOLAC in clinical practice; and v) perceived challenges regarding promoting TOLAC.

The first section was on sociodemographic factors and involved nine questions on sex, age, ethnicity, marital status, highest education level, type of hospital, professional title, administrative post, and duration of work experience (in years). The first section also involved the following question about the obstetrician’s own reproductive experience: *“Which mode of delivery have you/your wife experienced?”*. If the participant or spouse had undergone a CS, they were asked a Yes/No question on whether they intended to undergo TOLAC if they became pregnant again.

In the second section, the participants were asked the following Yes/No question on whether they intended to recommend TOLAC: *“Do you intend to recommend that pregnant women with a history of cesarean section undergo a trial of labor after cesarean if they meet the physiological criteria?*” In the third and fourth sections, two multiple-choice questions were used to investigate the obstetricians’ criteria for the selection of TOLAC in clinical settings and the basis underlying their selection criteria (the options were as follows: expert consensus in China, clinical experience, advice from superior physicians, textbook, and/or overseas guidelines), respectively. Finally, in the fifth section, there was a question on the perceived challenges regarding promoting TOLAC. The participants were asked to select as many factors as possible from a list of 11 items that they perceived as barriers to performing TOLAC.

After the first draft of the questionnaire was designed, the expert research team (four obstetricians, two midwives, two nurses, and two postgraduate students, three of whom had a PhD and four of whom had a master’s degree) gathered for a brainstorming session and reviewed and modified the questionnaire. Finally, everyone in the team rated their degree of agreement with each option of each item in the second section using a Likert scale, ranging from 1 = totally disagree to 4 = totally agree. High scores indicated a high level of compatibility. The average degree of expert authority was 0.91, and the significance level of Kendall’s W regarding the consistency of the expert opinions was < 0.05. Additionally, the questionnaire was tested by 12 obstetricians prior to initiating the study, and the pilot data showed that all 12 obstetricians understood the questionnaire and were able to complete it easily. Therefore, no changes were required prior to commencing the full study. Lastly, 30 obstetricians at three hospitals in our city were invited to fill in the questionnaire again after 4 weeks, and the test-retest correlation was 0.89.

### Statistical analyses

The survey data were exported from Questionnaire Star into SPSS (v20.0, IBM, USA) which was used for all data analysis. Descriptive statistics were used to present the demographic characteristics and study variables.

Differences in the rate of intention to recommend TOLAC by the obstetricians’ demographic characteristics (i.e., age, sex, ethnicity, highest education level, type of hospital, grade of hospital, professional title, administrative post, duration of work experience, marital status, and experience of vaginal delivery) and by perceived challenges regarding promoting TOLAC were assessed using a *t*-test (for normally distributed continuous variables) or a chi-square test (for categorical variables). Variables identified as being significant (*p* < 0.05) in these tests were then entered into a binary logistic regression model to determine the factors associated with the obstetricians’ intention to recommend TOLAC to pregnant women with a history of CS.

## Results

### Sociodemographic characteristics

Table 1 shows the sociodemographic information of the participants. The mean age of the participants was 36.8 ± 8.2 years; 89.0% of them were female, 85.0% were Han Chinese, and 85.5% were married. Nearly all of the obstetricians worked at general hospitals, and approximately a quarter held senior positions (Associate Senior Physician or Senior Physician) and had a graduate or higher degree. Overall, 39.2% of the obstetricians reported that they would choose TOLAC for themselves/their wife if they became pregnant and had a history of CS, while 69.0% intended to recommend TOLAC to pregnant women with a history of CS.

**Table 1:** Participant characteristics and associations between the demographics and intention to recommend TOLAC to pregnant women with a history of CS (*N* = 426)

	Total *mean* ± *SD/f (%)*	Intended (***n*** = 294) *mean (SD)/f (%)*	Not intended (***n*** = 132) *mean (SD)/f (%)*	***t/χ***^***2***^	***P***
**Age (years)**	36.76 ± 8.17	37.38 ± 8.38	35.36 ± 7.52	2.47	0.01*
**Sex**				0.74	0.41
Male	47 (11.0)	35 (11.9)	12 (9.1)		
Female	379 (89.0)	259 (88.1)	120 (90.9)		
**Ethnicity**				3.27	0.08
Han	362 (85.0)	256 (87.1)	(12.9)		
Minority	64 (15.0)	38 (80.3)	(19.7)		
**Highest education**				6.59	0.16
Technical secondary school	10 (2.3)	7 (2.4)	3 (2.3)		
Junior college	42 (9.9)	27 (9.2)	15 (13.0)		
Undergraduate university	255 (59.9)	171 (58.2)	84 (79.0)		
Postgraduate university	101 (23.7)	72 (24.5)	29 (31.3)		
Doctorate university	18 (4.2)	17 (5.8)	1 (5.6)		
**Type of hospital**				4.82	0.04*
General hospital	375 (88.0)	252 (85.7)	123 (93.2)		
Specialized hospitals of obstetrics and gynecology	51 (12.0)	42 (14.3)	9 (6.8)		
**Grade of hospital**^**&**^				0.01	1.00
Level 3	221 (51.9)	153 (52.0)	68 (51.5)		
Level 2	205 (48.1)	141 (48.0)	64 (48.5)		
**Professional title**				6.21	0.10
Resident physician	137 (40.6)	111 (37.8)	62 (47.0)		
Attending physician	140 (32.9)	98 (33.3)	42 (31.8)		
Associate senior physician	84 (19.7)	60 (20.4)	24 (18.2)		
Senior physician	29 (6.8)	25 (8.5)	4 (3.0)		
**Administrative post**				1.90	0.39
Director of obstetrics department	49 (11.5)	36 (12.2)	13 (9.8)		
Vice-director of obstetrics department	24 (5.6)	19 (6.5)	5 (7.4)		
None	253 (82.9)	239 (81.3)	114 (86.4)		
**Duration of work experience (year)**	12.93 ± 9.42	13.41 ± 9.63	11.87 ± 8.87	1.56	0.12
**Experienced vaginal delivery (themselves/their wife)**				4.76	0.03*
Yes	185 (43.4)	138 (46.9)	47 (35.6)		
No	241 (56.6)	156 (53.1)	85 (64.4)		
**Intention to choose TOLAC for themselves/their wife if they became pregnant and had a history of CS** ***(n = 148)***					
Yes	58 (39.2)	/	/		
No	90 (60.8)	/	/		

^&^*Hospitals in China are divided into three levels by hierarchical hospital management. Level 3 refers to hospitals with > 500 beds providing high-level medical care to several regions and undertaking higher education and research tasks. Level 2 refers to regional hospitals with 100–500 beds providing medical care to several communities and undertaking teaching and research tasks. Level 1 refers to primary hospitals with 20–99 beds directly providing integrated medical treatment, prevention, rehabilitation, and healthcare services to the* community

* p < 0.05 (χ^*2*^ *or t-test)*

### Selection criteria for TOLAC

More than 70.0% of the obstetricians reported that the selection criteria for TOLAC should include the following: “Patient agrees to TOLAC and understands the advantages and risks”, “Medical institutions have the resources and capacity (such as human resources, technology, and equipment) to deal with TOLAC complications”, “Fetus is in a cephalic dorsal position”, “Patient’s prior CS involved a transverse incision in the lower segment and no complications, and no contraindications for vaginal delivery exist in the present pregnancy”, “Parturient canal, fetus, force of labor, and patient’s mental factors are in a normal state”, “Ultrasonography shows that the muscular layer of anterior inferior uterus segment is in a normal state”, “Estimated fetal weight <3500 g”, and “No indications for cesarean section”. However, 56.1% of the obstetricians believed that the parturition interval (between the last CS and the present pregnancy) should be ≥24 months rather than ≥18 months and 6.6% considered that the estimated fetal weight should be < 4000 g rather than < 3500 g; these conservative precautions (regarding ≥24 months and < 4000 g) were not consistent with the expert consensus in China ***(***Table 2***).***

**Table 2:** Obstetricians’ criteria for selecting TOLAC in clinical settings (*N* = 426)

	***F***	%	Rank by frequency
Patient agrees to TOLAC and understands the advantages and risks	378	88.7	1
Medical institutions have the resources and capacity (such as human resources, technology, and equipment) to deal with TOLAC complications	369	86.6	2
Fetus is in a cephalic dorsal position	354	83.1	3
Patient’s prior CS involved a transverse incision in the lower segment and no complications, and no contraindications for vaginal delivery exist in the present pregnancy	350	82.2	4
Parturient canal, fetus, force of labor, and patient’s mental factors are in a normal state	338	79.3	5
Ultrasonography shows that the muscular layer of anterior inferior uterus segment is in a normal state	336	78.9	6
Estimated fetal weight < 3500 g	327	76.8	7
No indications for CS	312	73.2	8
Parturition interval ≥ 24 months	239	56.1	9
Parturition interval ≥ 18 months	102	23.9	10
Estimated fetal weight < 4000 g	28	6.6	11

The obstetricians’ selection criteria for TOLAC in clinical practice were mainly based on the expert consensus in China, clinical experience, and advice from superior physicians ***(***Table 3***)***.

**Table 3:** Basis underlying obstetricians’ selection criteria for TOLAC (N = 426)

	***f***	%	Rank by frequency
Expert consensus in China^%^	356	83.6	1
Clinical experience	311	73.0	2
Advice from superior physicians	234	54.9	3
Textbook	180	42.3	4
Overseas guidelines	97	22.8	5

^%^Expert consensus on vaginal delivery management of subsequent pregnancy after CS in 2016 in Chin a[25]

### Challenges regarding promoting/performing TOLAC

The obstetricians perceived that the main challenges regarding promoting/performing TOLAC were: lack of clear guidelines for predicting or avoiding the risks associated with TOLAC (83.4%); obstetricians’ uncertainty about the safety of TOLAC for women with a history of CS (81.2%), perceived unwillingness to accept the risks associated with TOLAC (81.0%) and perceived demand for ERCS (80.7%) among pregnant women; perceived lack of confidence (77.5%) or understanding (69.7%) regarding undergoing TOLAC among pregnant women and their families; worries about medical lawsuits due to adverse delivery outcomes (69.0%); and lack of clear acceptable medical standards or definitive guidelines for TOLAC in local clinical practice (55.9%) ***(***Table 4***).***

**Table 4:** Associations between perceived challenges regarding promoting/performing TOLAC and intention to recommend TOLAC to pregnant women with a history of CS among Chinese obstetricians (N = 426)

	Total *mean* ± *SD/f (%)*	Intended (n = 294) *mean (SD)/f (%)*	Not intended (n = 132) *mean (SD)/f (%)*	***t/χ***^***2***^	***P***
**Lack of clear guidelines for predicting or avoiding the risks associated with TOLAC, such as uterine rupture**				0.05	0.89
Yes	359 (84.3)	247 (84.0)	112 (84.8)		
No	67 (15.7)	47 (16.0)	20 (15.2)		
**Obstetricians’ uncertainty about the safety of TOLAC for pregnant women with a history of CS**				6.90	0.01*
Yes	346 (81.2)	229 (77.9)	117 (88.6)		
No	80 (18.8)	65 (22.1)	15 (11.4)		
**Perceived unwillingness to accept the risks associated with TOLAC among pregnant women with a history of CS**				1.20	0.29
Yes	345 (81.0)	234 (79.6)	111 (84.1)		
No	81 (19.0)	60 (20.4)	21 (15.9)		
**Perceived lack of confidence regarding undergoing TOLAC among pregnant women with a history of CS and their family members**				11.05	0.01**
Yes	330 (77.5)	241 (82.0)	89 (67.4)		
No	96 (22.5)	53 (18.0)	43 (32.6)		
**Perceived insufficient understanding or even misunderstanding regarding TOLAC among pregnant women with a history of CS and their family members**				0.48	0.50
Yes	297 (69.7)	208 (70.7)	89 (67.4)		
No	129 (30.3)	86 (29.3)	43 (32.6)		
**Worries about medical lawsuits due to adverse delivery outcomes after recommending TOLAC to pregnant women with a history of CS**				8.31	0.01**
Yes	294 (69.0)	194 (66.0)	100 (34.0)		
No	132 (31.0)	124 (93.9)	8 (6.1)		
**Lack of clear acceptable medical standards or definitive guidelines for TOLAC in local clinical practice**				1.00	0.32
Yes	238 (55.9)	169 (57.5)	69 (52.3)		
No	188 (44.1)	125 (42.5)	63 (47.7)		
**Lack of facilities to carry out TOLAC**				3.54	0.07
Yes	197 (46.2)	127 (43.2)	70 (53.0)		
No	229 (53.8)	167 (56.8)	62 (47.0)		
**Clinical experience and skill level of obstetrician are insufficient**				0.00	1.00
Yes	187 (43.9)	129 (43.9)	58 (43.9)		
No	239 (56.1)	165 (56.1)	74 (56.1)		
**Substandard gestation management of pregnant women with a history of CS**				0.26	0.67
Yes	182 (42.7)	128 (43.5)	54 (40.9)		
No	244 (57.3)	166 (56.5)	78 (59.1)		
**Clinical experience and skill level of midwife are insufficient**				0.94	0.39
Yes	163 (38.3)	108 (36.7)	55 (41.7)		
No	263 (61.7)	186 (63.3)	77 (81.5)		

* p < 0.05 (χ^*2*^ or t-test); ** p < 0.01 (χ^*2*^ *or t-test)*

### Factors associated with intention to recommend TOLAC to pregnant women with a history of CS

Differences in the rate of intention to recommend TOLAC to pregnant women with a history of CS between demographic subgroups of obstetricians, and the associations between perceived challenges regarding promoting TOLAC and intention to recommend TOLAC were assessed in univariate analyses. The following significant variables were then entered into a multivariate binary logistic regression model to identify the factors that were significantly associated with the intention to recommend TOLAC to pregnant women with a history of CS: age (*χ*^*2*^ = 2.47 *p* < 0.01); type of hospital (*χ*^*2*^ = 4.82, *p* = 0.04) ***(***Table 1***)***; perceived lack of confidence regarding undergoing TOLAC among pregnant women with a history of CS and their family members (*χ*^*2*^ = 4.76, *p* = 0.03); experienced vaginal delivery (themselves/their wife; *χ*^*2*^ = 11.05, *p* < 0.01); obstetricians’ uncertainty about the safety of TOLAC for pregnant women with a history of CS (*χ*^*2*^ = 6.90, p < 0.01); and worries about medical lawsuits due to adverse delivery outcomes (*χ*^*2*^ = 8.13, p < 0.01) ***(***Table 4***)***.

A best-fit binary logistic regression model (Omnibus Tests of Model Coefficients: χ^2^ = 64.32, p < 0.01; Hosmer–Lemeshow test: χ^2^ = 7.18, *p* = 0.52) identified the following factors as being significantly associated with intention to recommend TOLAC to women with a history of CS: perceived lack of confidence regarding undergoing TOLAC among pregnant women and their family members (OR = 2.31; 95% confidence interval [CI]: 1.38–1.38); obstetricians’ uncertainty about the safety of TOLAC for women with a history of CS (OR = 0.49; 95% CI: 0.27–0.96); and worries about medical lawsuits due to adverse delivery outcomes (OR = 0.14; 95% CI: 0.07–0.31) ***(***Table 5***).*** The results revealed that being uncertain about the safety of TOLAC for women with a history of CS and worries about medical lawsuits due to adverse delivery outcomes were the main factors effecting the intention to recommend TOLAC to women with a history of CS, while the perception that pregnant women and their family members lacked confidence about undergoing TOLAC was another contributing factor.

**Table 5:** Factors associated with an obstetrician’s intention on recommend TOLAC to a pregnant women with a prior CS (*N* = 381)

	Intendtion to recommend TOLAC	
	***OR (95% CI)***	***P***
**Age**	0.98 (0.95–1.01)	0.27
**Type of hospital**		
General hospital	1	
Specialized hospitals	1.96 (0.87–4.38)	0.10
**Experienced a vaginal delivery**		
No	1	
Yes	0.68 (0.41–1.13)	0.13
**Perceived lack of confidence regarding undergoing TOLAC among pregnant women with a history of CS and their family members**		
No	1	
Yes	2.31 (1.38–1.38)	0.01**
**Obstetricians’ uncertainty about the safety of TOLAC for pregnant women with a history of CS**		
No	1	
Yes	0.49 (0.27–.96)	0.04*
**Worries about medical lawsuits due to adverse delivery outcomes after recommending TOLAC to pregnant women with a history of CS**		
No	1	
Yes	0.14 (0.07–0.31)	0.01**

*a statistically significant adjusted odds ratio (AOR) with a p-value < 0.05;

**statistically significant AOR with a p-value < 0.01

## Discussion

This study revealed that two-thirds of obstetricians intend to recommend TOLAC to pregnant women with a history of CS. This proportion was lower than that reported in other studies [29, 31]. Doret et al. [31] found that all obstetricians from the Rhone-Alpes perinatal network in their study would like to offer VBAC to pregnant women who have experienced a single CS, as long as they do not have any contraindications for vaginal delivery. Sur et al. [29] found that 93% of obstetricians in two large UK medical deaneries would like to recommend TOLAC to all pregnant women with a history of CS if they have indications for vaginal delivery. The obstetricians’ intention to choose TOLAC for themselves/their wife if they became pregnant and had a history of CS provides a view on their perspective regarding this delivery mode. Our results revealed that only a third of the investigated obstetricians preferred TOLAC for themselves/their wife if they became pregnant and had a history of CS. In contrast, Sur et al. [29] found that 87% of obstetricians in their UK study preferred TOLAC if there were no labor contraindications. This reflects the fact that most Chinese obstetricians have a negative attitude about TOLAC. However, evidence has shown that physicians’ counseling/preference plays a critical role in pregnant women’s choice regarding TOLAC [32]. Consequently, it is necessary to pay close attention to obstetricians’ views on TOLAC and to help them to project a more positive attitude about TOLAC when discussing it with patients; this may improve the current low rate of TOLAC in China (9.3–13.0%) [13, 33].

The obstetricians considered multiple selection criteria when making a decision to offer TOLAC to pregnant women with a history of CS, including patient willingness to undergo TOLAC, medical conditions, fetal position, and involvement of a transverse incision in the lower segment and no complications in the patient’s prior CS. They also ensured that the parturient canal, fetus, force of labor, and patient’s mental condition were in a normal state, the muscular layer of the anterior inferior uterus segment was in a normal state, estimated fetal weight was < 3500 g, and there were no indications for CS. These findings are consistent with previous studies [28, 34, 35]. Although there are many different criteria for selecting patients for TOLAC, the obstetricians in our study mainly focused on patient willingness, indications for vaginal delivery, risk of complications, and the likelihood of successful VBAC.

Compared with selection criteria regarding selecting pregnant women with a history of CS for TOLAC on the expert consensus in China [27], the obstetricians’ selection criteria in practice tended to be conservative. For example, they would ensure that the parturition interval (between the last CS and the present pregnancy) was ≥24 months rather than ≥18 months; and the estimated fetal weight was < 3500 g rather than < 4000 g. The obstetricians’ tendency to be conservative in clinical practice might result from their wish to reduce or directly avoid the medical risks associated with TOLAC by strictly limiting the criteria for this delivery mode, combined with guidance from the expert consensus in China and their own practical experience [26].

In addition, we found that the obstetricians also took the advice of their superior physicians when making a decision regarding offering TOLAC to pregnant women with a history of CS. In China, a ward round must include three levels of physicians (senior physician or associate chief physician, attending physician, and resident physician). This is one of the basic medical care regulations in the Chinese medical system. This ensures that subordinate doctors can listen to the advice of senior doctors, as all doctors should be subordinate to the section chief when carrying out medical work [36]. Consequently, subordinate obstetricians should obey the decisions of their superior doctor when selecting delivery modes for pregnant women with a history of CS. Thus, the perceptions and attitude of superior physicians, particularly the chief physicians and associate chief physicians, play a significant role in decision-making regarding whether to offer TOLAC. Senior clinician training and education may have a large effect on encouraging senior doctors (and therefore junior doctors) in China to increase the TOLAC rate. This is important because obstetricians are concerned that there might be greater medical risks, or a medical dispute, if they recommend TOLAC to pregnant women with a history of CS. The obstetricians believed that lack of clear guidelines for predicting or avoiding the risks associated with TOLAC was the major challenge regarding performing TOLAC. Wells [28] reported that obstetricians who had never provided VBAC were unwilling to accept the risk of an adverse outcome, did not believe in the safety of VBAC, and wanted to avoid concerns related to medicolegal liability. Additionally, Cox [26] found that obstetricians’ fear of legal risk was the key reason to avoid VBAC and they preferred the convenience of an ERCS instead of having to remain in-house during a TOLAC. Kamal et al. [30] reported that obstetricians believe that clinical indications and medical evidence have a significant impact on decision-making when considering the delivery mode of pregnant women with a history of CS, and also stated that the main limitation to the options available was the perceived substandard quality of evidence in this area. Thus, multiple efficient strategies and policies, including comprehensively collecting the best evidence and devising medical standards, are now required to help obstetricians predict or avoid the medical risks associated with TOLAC.

Another factor that was closely associated with the obstetricians’ decision-making process was the perceived lack of confidence regarding undergoing TOLAC among some pregnant women and their family members. It is interesting that although most obstetricians considered this lack of confidence a challenge to promoting TOLAC, they would still like to encourage these pregnant women to select TOLAC. This phenomenon may reflect that the obstetricians’ concerns were the individual physical conditions and risk of complications rather than the women’s initial opinions regarding TOLAC.

The obstetricians also emphasized that pregnant women and their family members have insufficient knowledge of TOLAC, or have misunderstandings about the procedure, and may even ask for ERCS because of their concerns/lack of knowledge. Scaffidi et al. [37] found that if pregnant women had a high level of knowledge regarding the risks and benefits of TOLAC and ERCS, they were more likely to select TOLAC. It follows that taking measures to improve the level of knowledge among pregnant women with a history of CS and their families regarding the risks and benefits of TOLAC and ERCS is a key element for increasing the TOLAC rate.

Wong et al. [38] found that a novel one-stop obstetrician-led CS education and antenatal session increased the rate of VBAC selection by 38%. This involved inviting pregnant women with a history of CS to attend a 1-h discussion group facilitated by a consultant obstetrician. The women were provided with written information about the risks and benefits of VBAC and ERCS. The group discussed this information with the obstetrician, including their concerns and aspirations for their pregnancy and delivery. Knowledge regarding TOLAC should be popularized by this kind of session or other effective form of public health education to correct any misunderstandings, promote accurate knowledge, and enhance the profile of this delivery mode among the entire population, especially pregnant women with a history of CS.

Furthermore, approximately half of the obstetricians surveyed claimed that there was a lack of clear acceptable medical standards or definitive guidelines for TOLAC in local clinical practice. However, the United States, France, and Britain have published guidelines on TOLAC since 2010 [10, 20, 39], but these guidelines are not universally accepted by Chinese obstetricians. This might be due to the guidelines not taking clinical practice in China into account and so not appropriately guiding clinical practice in China. Moreover, a third of the obstetricians believed that the following factors can limit the practice of TOLAC: available facilities, clinical experience and skill levels of the local obstetrician and midwife, and substandard gestational management of pregnant women. Wanyonyi et al. [40] found that, among maternity service providers in East Africa, deficiencies in healthcare delivery systems, inadequate human resources, lack of unit guidelines, inappropriate maternal education, and inappropriate fetal monitoring tools were the main concerns regarding the practice of VBAC. Lazo-Porras et al. [41] conducted a qualitative prospective study to explore the decision-making process and final mode of delivery among pregnant women with a history of CS who were eligible for TOLAC; just 9 out of 17 participants stated that the physician explained that they could have chosen either TOLAC or ERCS for delivery and 6 participants did not receive any information from their providers about their delivery options. Authoritative standards are now required in China in order to promote TOLAC in clinical practice. These should include standards regarding the indications for TOLAC, labor management, and the prevention and management of complications.

### Limitations

This study has several limitations. First, this was an online study and the data collection relied upon self-reporting by the respondents, which may have resulted in selection and/or reporting bias. Additionally, we used a self-developed questionnaire, based on information in the published literature, and the validity of the questionnaire should be tested and discussed further. Furthermore, we adopted a cross-sectional study design to explore the topic, but further qualitative studies could obtain additional insights into Chinese obstetricians’ perspectives regarding TOLAC.

## Conclusions

This study has significant clinical utility for specialist professionals working in maternal and child healthcare in China and elsewhere. The findings from this study provide key evidence to help to create strategies for promoting TOLAC among women with a history of CS and to reduce the chance of blind decisions based on the current preference for ERCS rather than TOLAC/VBAC. There is a need for clear guidance and standards regarding indications for TOLAC, labor management, and the prevention and management of complications. There is a real need for public education and direct education for pregnant women with a history of CS to improve decision-making between patients and their doctors regarding adopting TOLAC in clinical practice.

## Supplementary Information

**Additional file 1.**

## Data Availability

The dataset analyzed for the current study is available from the corresponding author on reasonable request.

## Abbreviations

TOLAC: Trial of labor after cesarean
CS: Cesarean section
ERCS: Elective repeat cesarean section
VBAC: Vaginal birth after cesarean
NHS: National Health Commission
