# Supplementary material for: Obstetricians’ perspectives on trial of labor after cesarean (TOLAC) under the two-child policy in China: a cross-sectional study
Source: BMC Pregnancy Childbirth. 2021 Jan 28;21:89. doi: 10.1186/s12884-021-03559-1 (PMC7841882; doi:10.1186/s12884-021-03559-1)
Supplement: Supplementary file 1 — Additional file 1. [file 12884_2021_3559_MOESM1_ESM.doc]

**Questionnaires regarding obstetricians' perspectives on trial of labor after cesarean under the two-child policy in China**

Dear obstetrician:

Greeting! We conduct this anonymous questionnaires in order to understand the obstetrician's attitude toward trial of labor after cesarean under the adjustment of the birth policy (the two-child policy in China). If you would like to participate in this survey, then please read each question bellow carefully and fill in your real situation on the following‘______’, or choose what do you think is the most appropriate answer in its corresponding‘□’. We will keep the information you provide strictly confidential, and this survey will not cause any adverse effects on your. Thanks for your support and cooperation.

- Are you a clinical obstetrician with a qualification granted by the National Health Commission (NHC)?

^1^□Yes ^2^□No

- Are you working in a gynecology and obstetrics department that allows a trial of labor after cesarean to be offered to women with a history of cesarean section?

^1^□Yes ^2^□No

**Characteristics**

1.**Sex**:^1^□Male ^2^□Female

2.**Age**: (years)

3.**Ethnicity**:^1^□Han ^2^□Minority

4. **Marital status**:^1^□Single ^2^□Married

5.**Highest** **education**:^1^□ Technical secondary school ^2^□Junior college ^3^□Undergraduate university

^4^□Postgraduate university ^5^□Doctorate university

6.**Type of your hospital**

^1^□General hospital ^2^□Specialized hospitals of obstetrics and gynecology ^3^□Others__________

7.**Grade of your hospital**

^1^□ Level 3 hospital ^2^□ Level 2 hospital

8.**Professional Title**:^1^□Resident physician ^2^□Attending physician ^3^□Associate senior physician ^4^□Senior physician

9.**Administrative post**:^1^□Director of obstetrics department ^2^□Vice-director of obstetrics department ^3^□None

10.**Duration of work experience**: (years)

11.**Which mode of delivery have you/your wife experienced?**

^1^□None ^2^□Only cesarean-section ^3^□Only vaginal delivery

^4^□ Cesarean-section after vaginal delivery ^5^□Vaginal birth after cesarean section

**12.Do you intent to choose TOLAC for yourself/your wife if yourself/your wife became pregnant and had a history of** **cesarean section?**

^1^□Yes  ^2^□No

**13.Do you intend to recommend that pregnant women with a history of cesarean section undergo a trial of labor after cesarean if they meet the physiological criteria?**

^1^□Yes  ^2^□No

**14.Which are the selection criteria (indications) for trial of labor after cesarean in your clinical practice?**

|  | **Events** | **Yes** | **No** |
| --- | --- | --- | --- |
| 1 | Medical institutions have the resources and capacity (such as human resources, technology, and equipment) to deal with TOLAC complications |  |  |
| 2 | Patient’s prior CS involved a transverse incision in the lower segment and no complications, and no contraindications for vaginal delivery exist in the present pregnancy |  |  |
| 3 | Fetus is in a cephalic dorsal position |  |  |
| 4 | Parturient canal, fetus, force of labor, and patient’s mental factors are in a normal state |  |  |
| 5 | Ultrasonography shows that the muscular layer of anterior inferior uterus segment is in a normal state |  |  |
| 6 | Estimated fetal weight <3500 g |  |  |
| 7 | Estimated fetal weight <4000 g |  |  |
| 8 | Parturition interval ≥18 months |  |  |
| 9 | Parturition interval ≥24 months |  |  |
| 10 | Patient agrees to TOLAC and understands the advantages and risks |  |  |
| 11 | No indications for CS |  |  |
| 12 | Others (please write it done) | | |

**15.Which is the basis underlying the selection criteria for trial of labor after cesarean in your clinical practice?**

|  | **Events** | **Yes** | **No** |
| --- | --- | --- | --- |
| 1 | Expert consensus in China% |  |  |
| 2 | Clinical experience |  |  |
| 3 | Advice from superior physicians |  |  |
| 4 | Textbook |  |  |
| 5 | Overseas guidelines |  |  |
| %Expert consensus on vaginal delivery management of re-pregnancy after cesarean section in 2016 in China. | | | |

**15.What are the challenges regarding promoting the trial of labor after cesarean in your opinion?**

|  | **Events** | **Yes** | **No** |
| --- | --- | --- | --- |
| 1 | Lack of facilities to carry out TOLAC |  |  |
| 2 | Clinical experience and skill level of obstetrician are insufficient |  |  |
| 3 | Clinical experience and skill level of midwife are insufficient |  |  |
| 4 | Substandard gestation management of pregnant women with a history of CS |  |  |
| 5 | Obstetricians’ uncertainty about the safety of TOLAC for pregnant women with a history of CS |  |  |
| 6 | Lack of confidence regarding undergoing TOLAC among pregnant women with a history of CS and their family members |  |  |
| 7 | Unwillingness to accept the risks associated with TOLAC among pregnant women with a history of CS |  |  |
| 8 | Insufficient understanding or even misunderstanding regarding TOLAC among pregnant women with a history of CS and their family members |  |  |
| 9 | Lack of clear guidelines for predicting or avoiding the risks associated with TOLAC, such as uterine rupture |  |  |
| 10 | Worries about medical lawsuits due to adverse delivery outcomes after recommending TOLAC to pregnant women with a history of CS |  |  |
| 11 | Lack of clear acceptable medical standards or definitive guidelines for TOLAC in local clinical practice |  |  |
| 12 | Others (please write it done) | | |
